# A Subcellular Keap1-Nrf2 Disruption Strategy for Atopic Dermatitis Therapy

**DOI:** 10.1021/acs.jmedchem.6c00772

**Published:** 2026-08-21

**Authors:** Zixi Zhang, Jia Wu, Jingqi Liu, Daniel Shiu-Hin Chan, Chun-Yuen Wong, Jing Wang, Chung-Hang Leung, Wanhe Wang

**Affiliations:** † School of Life Science and Technology, 26487 Northwestern Polytechnical University, 1 Dongxiang Road, Xi’an, Shaanxi 710072, China; ‡ State Key Laboratory of Mechanism and Quality of Chinese Medicine, 59193 University of Macau, Taipa, Macao SAR 999078, China; § Institute of Chinese Medical Sciences, University of Macau, Taipa, Macao SAR 999078, China; ∥ Research & Development Institute of Northwestern Polytechnical University in Shenzhen, 45 South Gaoxin Road, Shenzhen, Guangdong 518057, China; ⊥ Department of Chemistry, 2152 City University of Hong Kong, Kowloon, Hong Kong 999077, China; ¶ College of Chemistry and Materials Science, State Key Laboratory of Bioactive Molecules and Druggability Assessment, Jinan University, Guangzhou, 510632, China

## Abstract

Nuclear factor erythroid 2-related factor 2 (Nrf2) is a promising target for atopic dermatitis due to its role in skin barrier repair, yet effective modulators of Nrf2 are lacking. Here, we report an itaconate-iridium­(III) complex conjugation strategy for the disruption of the Keap1-Nrf2 protein–protein interaction (PPI) for treating atopic dermatitis. The conjugate (**1**) specifically engages cytoplasmic Keap1 to restore Nrf2 activity, thus upregulating anti-inflammatory genes HO-1 and NQO1 while suppressing reactive oxygen species (ROS) in keratinocytes. It also reduces IL-13 production and boosts skin barrier proteins filaggrin and involucrin in a reconstructed human epidermis (RHE) model. *In vivo* studies revealed that compound **1** ameliorated DNFB-induced atopic dermatitis-like lesions and hypersensitivity. Its optical characteristics further enabled the flow cytometric discrimination of HaCaT cells based on Keap1 levels. This work provides a promising strategy for developing bioactive molecules to efficiently disrupt subcellular PPIs, laying the basis to progress theranostic candidates for atopic dermatitis.

## Graphical abstract

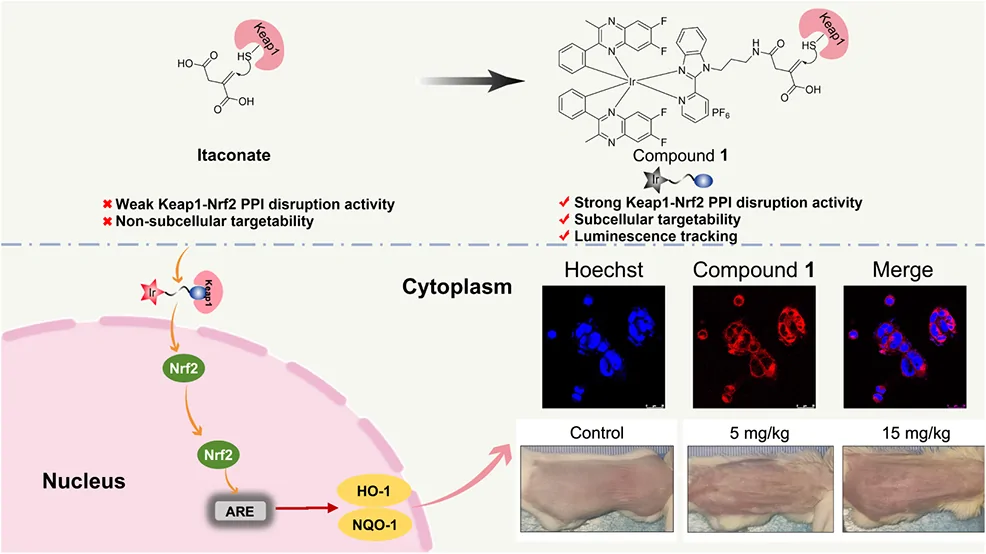

## Introduction

Atopic dermatitis is a commonly occurring chronic recurrent inflammatory skin disorder that is characterized by itchy, red, swollen, and cracked skin, compromising the qualify life of patients.^1^ Risk factors include a defective skin barrier, interleukin (IL)-4/IL-13 axis inflammation, type 2 immunity, genetic predispositions, and other causative environmental factors.^2,3^ Current atopic dermatitis treatment mainly relies on the blockage of the JAK/STAT pathway or direct inhibition of the IL-4 receptor (IL-4R) to decrease the pro-inflammatory effects of IL-4 and IL-13.^4^ In 2020, Sanofi developed dupilumab, the world’s first biologic agent targeting IL-4Rα, to treat moderate and severe atopic dermatitis.^5^ However, monoclonal antibodies are still limited by high cost and complicated intake methods, and/or elusive therapeutic mechanisms.^6^ Meanwhile, current oral and topical small-molecule drugs also suffer from a variety of safety issues. The adverse effects associated with these treatments underscore the critical need for developing safer and more effective therapeutic compounds.

Apart from targeting JAK or IL-4Rα, skin cells defend against atopic dermatitis-associated inflammatory responses by activating nuclear respiratory factor-2 (Nrf2), a key component that helps to regulate the redox equilibrium of cells.^7^ Under normal conditions, Nrf2 levels in the cytoplasm are low as a result of being ubiquitinated and degraded under the influence of Kelch-like ECH-associated protein (Keap1).^8^ However, oxidizing agents can convert Keap1’s cysteine residues into disulfide groups and conjugate them to electrophiles. This results in the dissociation of Nrf2, its nuclear translocation, and the subsequent upregulation of antioxidant genes in keratinocytes.^7,9^ Increasing evidence has demonstrated the critical actions of Keap1-Nrf2 in epidermal keratinization, supporting its therapeutic role in preventing atopic dermatitis.^10^ Upon activation of Nrf2, IL-4 and IL-13 will be induced to bind to receptors on the surface of keratinocytes, inducing the expression of skin barrier proteins.^11^ Moreover, activated Nrf2 can upregulate antioxidative enzymes such as NAD­(P)H dehydrogenase [quinone] 1 (NQO1), which protect vascular endothelial cells from harm induced by reactive oxygen species (ROS).^12^ Therefore, enhancing Nrf2 activity is considered as a potential strategy to lower ROS levels, while upregulating essential skin barrier proteins filaggrin and involucrin for combating atopic dermatitis.^13^

Current strategies for enhancing Nrf2 activity mainly rely on the inhibition of Keap1.^14^ Over the past decade, a number of Keap1 antagonists have been described, and most of them target cysteine residues in Keap1 covalently.^15^ In particular, Keap1-Nrf2 protein–protein interaction (PPI) inhibitors have arisen as a novel type of Nrf2 activators for treating acetaminophen-induced acute liver injury and alcoholic liver disease.^16,17^ Despite this, the potential of Keap1-Nrf2 PPI inhibitors for atopic dermatitis therapy remains unexplored due to the lack of highly potent PPI molecules.

Itaconate, a metabolite from the Krebs cycle, contains an electrophilic α,β-unsaturated carboxylic acid that can take part in alkylation reactions with Keap1 protein cysteine residues via Michael addition to form a 2,3-dicarboxypropyl species.^18^ Thus, it can disrupt the Keap1-Nrf2 PPI, resulting in the accumulation of transcriptionally active Nrf2, which subsequently migrates to the nucleus to enhance antioxidant and anti-inflammatory gene expression.^18^ Itaconate has shown the ability to modulate type I interferon responses through Nrf2 activation, and derivatives of itaconate also exhibited a positive effect on the uptake of fibrous type I collagen and wound healing.^19,20^ However, due to the small size of itaconate and its derivatives, they showed relatively low Keap1-Nrf2 PPI disruption activity, compromising their anti-inflammatory and skin-protective capabilities for treating atopic dermatitis.

The enrichment of bioactive molecules at subcellular locations can effectively regulate intracellular processes.^21^ Our previous work demonstrates that the conjugation of octahedral transition-metal complexes such as iridium­(III) promotes subcellular accumulation of bioactive molecules, while the increased steric size of conjugates provides effective PPI disruption activity, amplifying the biological activity of bioactive molecules.^22–24^ Based on this, we hypothesized that isocyanate-functionalized iridium­(III) complexes could efficiently bind subcellular Keap1 and disrupt the Keap1-Nrf2 PPI for activating Nrf2 to treat atopic dermatitis.

Herein, we developed a luminescent iridium­(III) complex-conjugated itaconate (**1**), which enhances the potency of itaconate through specifically disrupting the cytoplasmic Keap1-Nrf2 PPI (Figures 1A and B). In human keratinocyte HaCaT cells, compound **1** raised the transcription of heme oxygenase-1 (HO-1) and NQO1, with higher efficacy than the well-known Keap1-Nrf2 PPI inhibitor ML334.^8^ It inhibited the production of the pro-inflammatory cytokine IL-13 and increased the levels of skin barrier proteins filaggrin and involucrin in a reconstructed human epidermis (RHE) atopic dermatitis model. The *in vivo* efficacy of compound **1** was validated in a DNFB-induced atopic dermatitis mouse model, demonstrating its capacity to alleviate skin inflammation and hypersensitivity with a favorable biosafety profile compared to dexamethasone. Moreover, its inherent luminescence enables the direct visualization of its subcellular localization, eliminating the need to incorporate an additional fluorescent probe. Compound **1** showed bright luminescence in keratinocyte HaCaT skin cells but low luminescence in A375 skin cancer cells, demonstrating its clinical potential as an imaging agent for delineating atopic dermatitis margins and guiding precise tissue excision. These combined capabilities establish compound **1** as the first theranostic agent for imaging and targeting the Keap1-Nrf2 pathway for atopic dermatitis.

**Figure 1:**
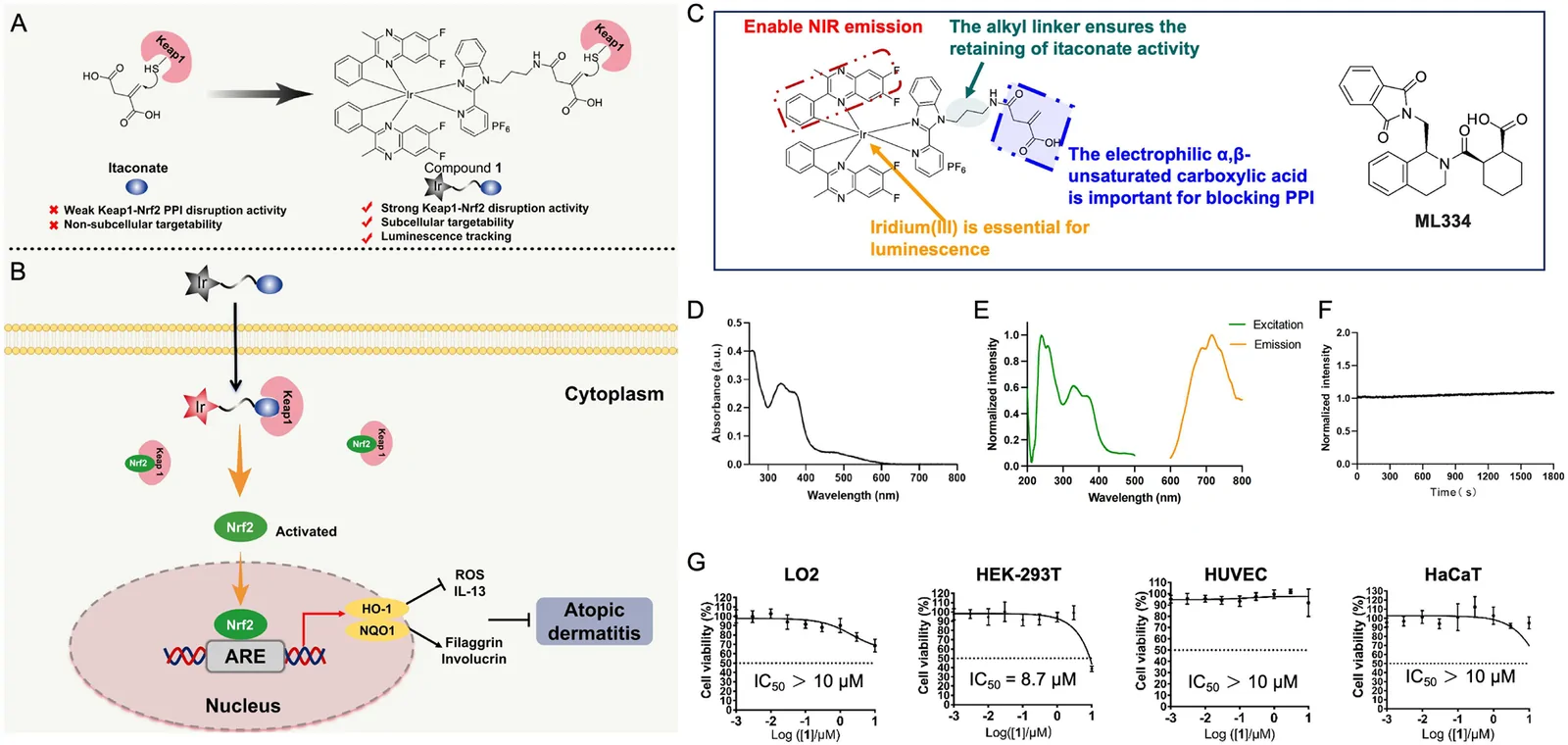
Schematic illustration of compound **1** as a Keap1-Nrf2 PPI inhibitor for treating atopic dermatitis. (A) Principle of the design of compound **1**. (B) Mechanistic diagram of compound **1** for targeting Keap1-Nrf2 PPI in HaCaT cells for treating atopic dermatitis. (C) Design of compound **1** and the chemical structure of ML334. (D) Absorption spectra of 10 μM compound **1** in PBS. (E) Excitation and emission spectra of 10 μM compound **1** in PBS. (F) Time course of luminescence of compound **1** (10 μM) in DMSO over 1800 s under continuous irradiation at 365 nm. (G) Cytotoxicity of compound **1** in different cell lines after 72 h. Cells were treated with 0–10 μM of compound **1** for 72 h, and cytotoxicity was assessed using the MTT assay.

## Results

### Design and Synthesis of Compound **1** as a Keap1-Nrf2 Interaction Inhibitor

Itaconate has been identified as a disruptor of the Keap1-Nrf2 PPI. It acts through alkylation of Keap1 protein’s cysteine residues through its electrophilic α,β-unsaturated carboxylic acid group by a Michael addition reaction.^18^ However, its activity for disrupting Keap1-Nrf2 PPI is too weak to be a candidate drug for atopic dermatitis. Given that iridium­(III) complexes offer the characteristics of subcellular targeting potential, inherent luminescence, and selective interactions with biomolecules, we hypothesized that conjugating a luminescent iridium­(III) complex with itaconate could enhance its inhibitory activity against the Keap1-Nrf2 PPI (Figure 1C). Cyclometalating ligands bearing a fluorine atom are known to modulate the optical properties and subcellular distribution of iridium­(III) complexes. Therefore, the widely used 6,7-difluoro-2-methyl-3-phenylquinoxaline (dfpq) ligand in our lab was selected. This ligand features extended π-conjugation, enabling red-shifted emission of over 650 nm, while its lipophilicity facilitates cellular uptake and cytoplasmic localization.^25,26^ Meanwhile, the ease of derivation of the 2-(2-pyridyl)-benzimidazole-type ancillary ligand prompted us to use it to connect the terminal acid site of itaconate through an alkyl linker, as this acid site has been identified to be tolerable to modification.^27^ Finally, the electrophilic α,β-unsaturated carboxylic acid group of the itaconate ligand is critical for targeting the Keap1-Nrf2 PPI (Figure 1C).

Compound **1** was synthesized by a straightforward pathway (Scheme S1): 2-(2-pyridyl)-benzimidazole was first substituted by *N*-boc-3-aminopropyl bromide with potassium hydroxide (KOH) to obtain compound **S1**, which was deprotected by hydrochloric acid (HCl) to obtain intermediate **S2**. The resultant **S2** was then coupled by monobutyl itaconate with the assistance of 1-ethyl-3-(3-dimethylaminopropyl)­carbodiimide hydrochloride (EDCI) and 1-hydroxybenzotriazole (HOBt) to afford intermediate **S3**, which was then hydrolyzed by lithium hydroxide (LiOH) to generate ligand **S4**. The final ligand was coordinated with the chloride-bridged dimer Ir_2_(dfpq)_4_Cl_2_ to give the desired conjugate **1**. Compound **2** without the itaconate moiety was also prepared according to our previous report (Scheme S2).^28^ All intermediates and final compounds were fully characterized by electrospray ionization mass spectrometry (ESI-MS)/high-resolution mass spectrometry (HRMS), ^1^H NMR, and ^13^C NMR (Figures S1–S7). The purity of compound **1** was assessed by high-performance liquid chromatography (HPLC) to have a purity of over 95% (Figure S8), sufficient for further biological evaluations.

The optical characteristics of the complex were studied by absorption spectra and luminescence spectra in phosphate-buffered saline (PBS) solution (Table S1). Compound **1** exhibits a strong absorption band at about 310–400 nm, presumably due to the spin-allowed metal–ligand charge transfer (^1^MLCT) transition, and a relatively weak absorption band from 430 to 600 nm ascribed to a typical ^3^MLCT transition (Figure 1D).^29,30^ The excitation spectrum exhibited a similar pattern to the absorption spectrum. The luminescence emission spectrum showed a maximum emission at 710 nm, indicating a wide Stokes shift of 230 nm between ^3^MLCT absorption and emission, beneficial for avoiding the interference of the excitation source (Figure 1E). Moreover, the near-infrared (NIR) emission of compound **1** can offer better imaging performance with less autofluorescence interference in biological systems. Furthermore, compound **1** has a long emission time of 37.73 ns in PBS buffer (Table S1) and exhibited negligible luminescence variation even under 1800 s continuous irradiation at 365 nm (Figure 1F), consistent with the high photostability of iridium­(III) complexes.

### Compound **1** Displays Low Cytotoxicity to Cells

Compound **1**’s cytotoxicity was evaluated in a range of human normal cell lines, including the liver cell line LO2, the human umbilical vein endothelial cell line HUVEC, the human embryonic kidney cell line HEK293T, and the human keratinocyte cell line HaCaT. Compound **1** had no significant cytotoxicity in LO2 (IC_50_ > 10 μM), HUVEC (IC_50_ > 10 μM), and HaCaT (IC_50_ > 10 μM) after 72 h, except for slight cytotoxicity in HEK293T (IC_50_ = 8.7 μM) (Figure 1G). These indicated that compound **1** was potentially biocompatible for treating atopic dermatitis.

### Compound **1** Engages Keap1 *In Cellulo*

HaCaT cells are the most commonly used cell model for studying epidermal homeostasis and pathophysiology.^31^ The cellular thermal shift assay (CETSA) was performed in HaCaT cell lysates to assess the engagement of compound **1** to Keap1 and Nrf2. Compound **1** significantly stabilized Keap1 even at high temperatures up to 70 °C, whereas no significant stabilized effect was observed for Nrf2 (Figures 2A and B). Hence, compound **1** can target Keap1 in cell contexts. Human recombinant Keap1 protein was purified from *E. coli* (BL21) (Figure S9). An isothermal titration calorimetry (ITC) experiment was performed to assess the thermodynamic characteristics of compound **1** binding to Keap1. Compound **1** bound to Keap1 with an approximate 2:1 stoichiometry (*N* = 1.95 ± 0.12) and a dissociation constant of *K*_d_ = 0.627 ± 0.282 μM (Figure 2C). Additionally, this interaction between compound **1** and Keap1 could be influenced by enthalpy/entropy compensation (Δ*G* = −8.46 kcal/mol, Δ*H* = −12.2 ± 1.0 kcal/mol), which suggests that reversible interactions could have an important role in binding. Liquid chromatography mass spectrometry (LC-MS) was further conducted to directly probe the interaction between compound **1** and Keap1. The results showed the appearance of a new peak with a mass shift that matched the theoretical value of the covalent adduct between compound **1** and Keap1 (Figure S10). This result suggests that that compound **1** covalently binds to the critical cysteine residues of Keap1 through its electrophilic itaconate moiety. The discrepancy between ITC and LC-MS results may arise from the fact that the binding process between compound **1** and Keap1 follows a two-step mechanism: initial reversible noncovalent docking, followed by subsequent specific covalent modification. To further verify that compound **1** interacts with Keap1 protein in human keratinocytes, an imaging experiment was conducted in HaCaT cells with pretreatment of Keap1 siRNA. The luminescence of compound **1** was diminished in the Keap1 knockdown group relative to the control siRNA-treated group (Figures 2D and E), providing evidence that compound **1** binds to Keap1 in the cytosol to enhance the emission signal. To further verify the target of compound **1**, we performed a competition experiment with free itaconate. Preincubation with itaconate (1 and 3 μM) resulted in a dose-dependent decrease of the luminescence signal of compound **1** (Figure 2F), supporting the mechanism that compound **1** targets Keap1 through its itaconate moiety. In addition, compound **1** (10 μM) exhibited no luminescence response to common cellular species in PBS, such as amino acids, anions, metal ions, and protein bovine serum albumin (BSA), highlighting the specific binding of compound **1** to Keap1 (Figure S11).

**Figure 2:**
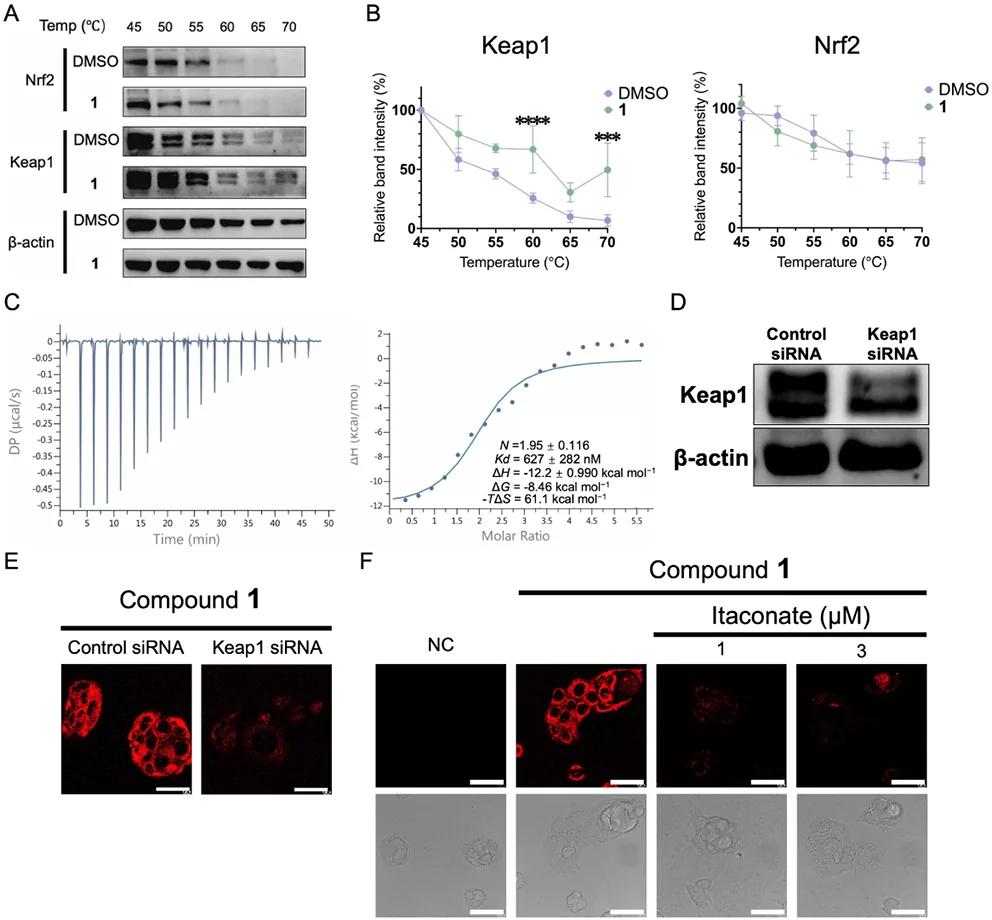
Compound **1** engages Keap1 *in cellulo*. (A) Thermal stabilization of Keap1 and Nrf2 by compound **1** and densitometry analysis. Proteins were detected by Western blotting using the corresponding antibodies. (B) Quantification analysis of Keap1 and Nrf2 in Western blotting. Data are represented as mean ± SD. *p < 0.05 vs DMSO group (Student’s *t* test). (C) ITC thermograms showing titration of 150 μM of compound **1** into Keap1 (5 μM). (D) Western blot assay for Keap1 protein levels in HaCaT cells with or without Keap1 siRNA treatment. (E) Effects of Keap1 knockdown on the imaging of compound **1** (10 μM, 2 h, λ_ex_/λ_em_ = 405/600–750 nm) in HaCaT cells. Scale bar = 50 μm. (F) Luminescence imaging of compound **1** in HaCaT cells. Cells were treated with or without 1 μM and 3 μM itaconate for 4 h and then added 10 μM compound **1** for 2 h. λ_ex_/λ_em_ = 405/600–750 nm. Scale bar = 50 μm.

### Compound **1** Specifically Inhibits Cytoplasmic Nrf2-Keap1 PPI and Promotes Nuclear Accumulation *In Cellulo*

Based on the findings above, we hypothesized that compound **1** is capable of entering the cytoplasm of HaCaT cells, disrupting the Keap1-Nrf2 PPI, and stimulating the movement of free Nrf2 protein into the nucleus, which in turn activates antioxidant genes (Figure 3A). By utilizing the inherent luminescence of compound **1**, its uptake and distribution in HaCaT cells was directly monitored by live cell imaging, along with the nuclear dye Hoechst. The results revealed that compound **1** mainly accumulated in the cytoplasm (Figure 3B), consistent with its ability to engage Keap1. The uptake of compound **1** was also studied by inductively coupled plasma mass spectrometry (ICP-MS), which revealed that compound **1** effectively entered HaCaT cells at 1.5 h, with slightly higher uptake at 2.5 h, indicating good cellular permeability (Figure 3C). Subsequently, the subcellular fractions were analyzed by ICP-MS. Compound **1** was found to be predominantly distributed in the cytoplasm after 2 h treatment, consistent with the luminescence imaging results (Figures 3D and S12). Fluorescence polarization (FP) assay is a common screening tool for detecting PPI inhibitors *in vitro*, as it can assess the PPI disruption ability of molecules. A fluorescently labeled Nrf2 peptide bearing the high-affinity ETGE motif was used as a fluorescent tracer to detect disruption of the Keap1-Nrf2 interaction. The FP result showed that compound **1** can efficiently disrupt the Keap1-Nrf2 interaction, with an EC_50_ of 14.2 μM (Figure 3E).

**Figure 3:**
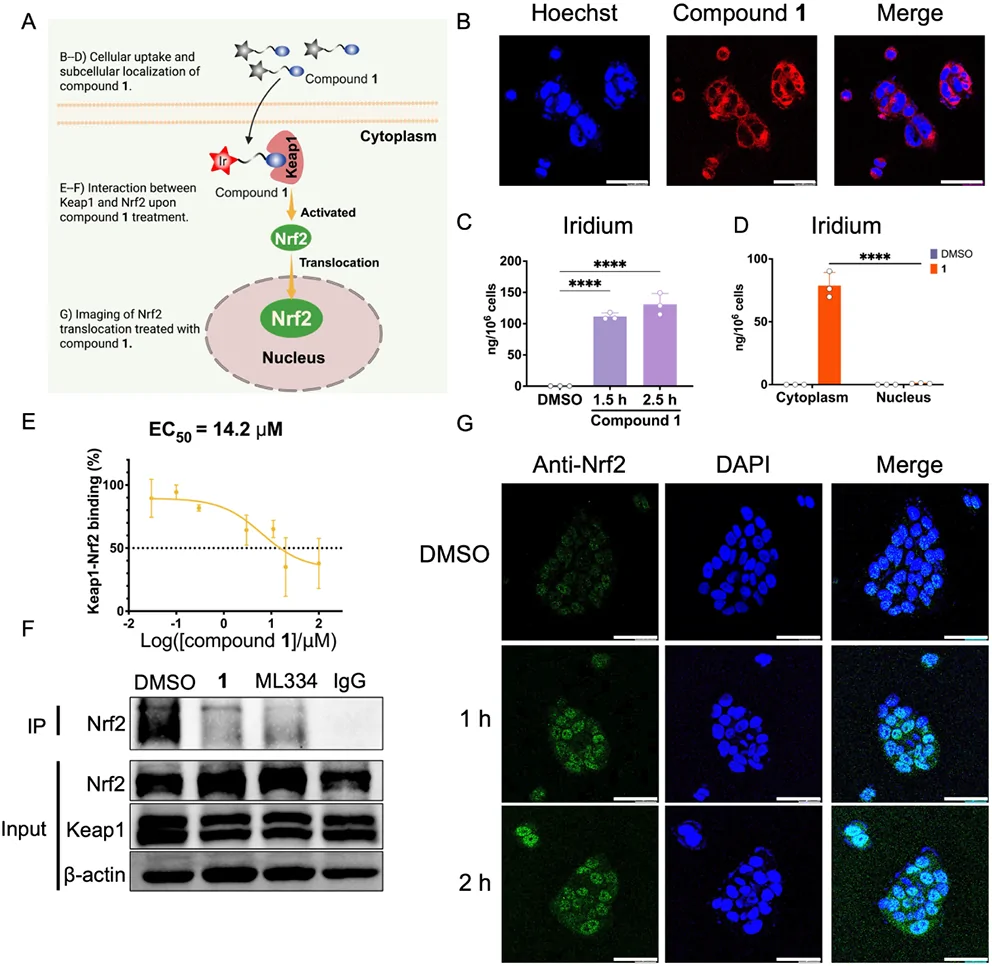
Cellular permeability of compound **1** and its effect on the Keap1-Nrf2 PPI and Nrf2 translocation. (A) Schematic diagram of the mechanism of action of compound **1** against the Keap1-Nrf2 PPI. (B) Confocal images of HaCaT cells pretreated with compound **1** (10 μM, red, λ_ex_/λ_em_ = 405/600–750 nm) for 2 h, followed by staining with Hoechst (blue) for 0.5 h. Scale bar = 50 μm (C) ICP-MS quantification of cell uptake for compound **1**. HaCaT cells were incubated with 10 μM compound **1** for 1.5 or 2.5 h. Error bars represent the standard deviations of the results from three independent experiments. (D) ICP-MS quantification of compound **1** in subcellular fractions. HaCaT cells were incubated with 10 μM compound **1** for 2 h. Error bars represent the standard deviations of the results from three independent experiments. P values were calculated using a one-way ANOVA with Tukey’s multiple comparison test. ****p < 0.0001 vs control group. (E) Dose–response effect of compound **1** on Keap1-Nrf2 binding via an FP assay. (F) Interactions between Keap1 and Nrf2 in HaCaT cells were examined by Western blotting and co-IP. This experiment was repeated twice with similar results. (G) Immunofluorescence image of Nrf2 (green, λ_ex_/λ_em_ = 488/500–600 nm) in HaCaT cells treated with compound **1**, compared to its predominantly cytoplasmic localization in untreated control cells. Nuclei are counterstained with DAPI (blue, λ_ex_/λ_em_ = 405/400–500 nm). Scale bar = 50 μm.

A co-immunoprecipitation assay was further performed to assess the capability of compound **1** to disrupt Keap1-Nrf2 in HaCaT cells. Compound **1** decreased the amount of Nrf2 coprecipitated with Keap1, confirming that the compound disrupts the Keap1-Nrf2 interaction *in cellulo* (Figure 3F). A further immunofluorescence imaging experiment showed that compound **1** enhanced the levels of nuclear Nrf2 in HaCaT cells after 1 h of treatment (Figure 3G). This observation is consistent with the mechanism whereby Keap1-Nrf2 inhibition after an oxidant challenge facilitates the movement of released Nrf2 to the nucleus. Taken together, compound **1** is capable of specifically disrupting the cytoplasmic Keap1-Nrf2 interaction, thereby activating Nrf2 nuclear translocation.

### Compound **1** Activates the Antioxidative Process via Blocking the Keap1-Nrf2 Interaction

Nuclear Nrf2 forms heterodimers that recognize ARE, thus activating antioxidant proteins, including HO-1 and NQO1, resulting in a decrease of ROS levels to preserve oxidative balance and protect the cell from oxidative stress (Figure 4A).^9,32^ The HaCaT cell line mimics the impaired antioxidant response reported in atopic dermatitis.^31^ Western blotting experiments showed that treatment of HaCaT cells with Keap1 siRNA elevated Nrf2 levels, consistent with the inhibitory role of Keap1 on Nrf2 protein levels. Additionally, compound **1** was less effective at increasing Nrf2 levels in Keap1 knockdown cells compared with control cells, further supporting Keap1 as a target of compound **1** (Figures 4B and C). A dose experiment showed that compound **1** could induce NQO1 and HO-1 protein levels in a dose-dependent manner (1–10 μM) (Figures 4D and E). Hence, compound **1** increases NQO1 and HO-1 protein levels via regulating Keap1-Nrf2 interaction in HaCaT cells. ARE transcriptional activity was then measured after treating HaCaT cells with compound **1** (10 μM) using the dual luciferase assay, with results being compared to compound **2**, itaconate, and ML334. Compound **1** activated higher ARE transcriptional activity than itaconate and ML334 under the same conditions, while compound **2** without the itaconate moiety induced minimal ARE transcriptional activity (Figure 4F). Overall, compound **1** effectively stimulates the Nrf2 anti-inflammatory pathway through the synergistic integration of the iridium­(III) complex and itaconate.

**Figure 4:**
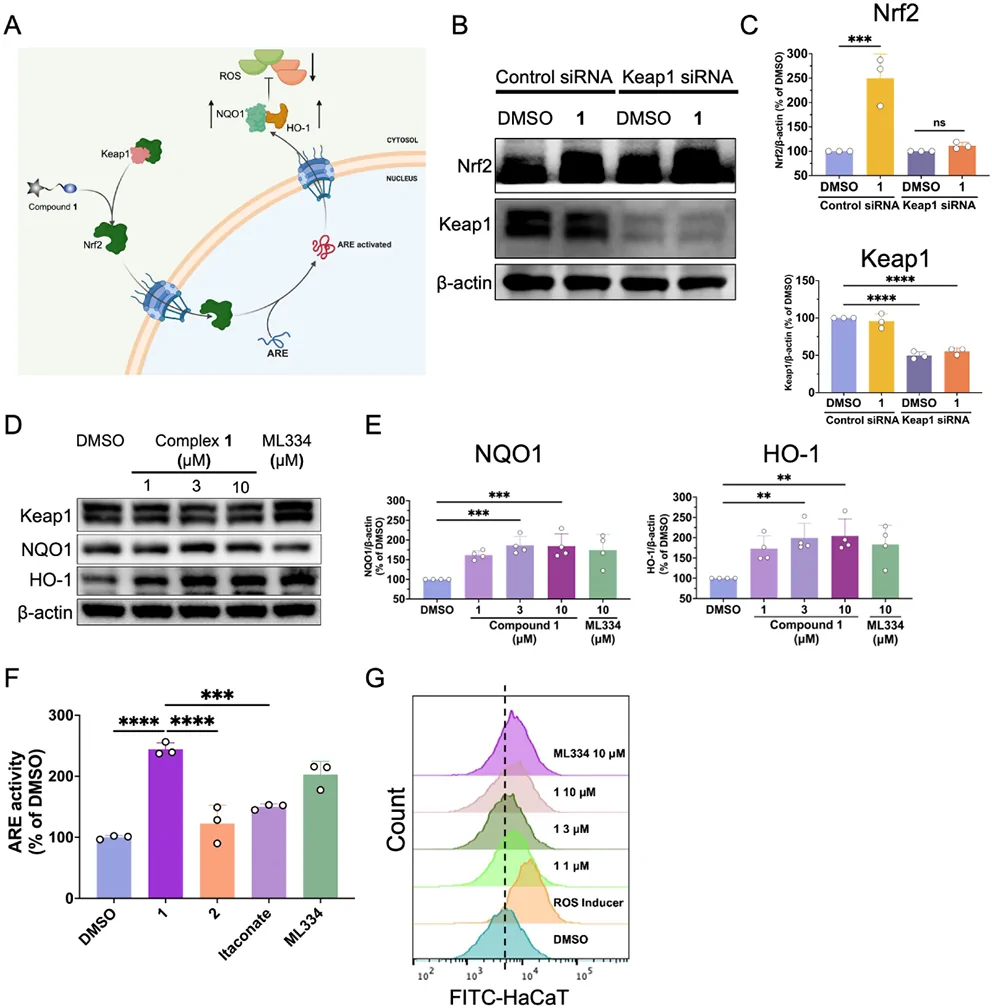
Compound **1** activates antioxidative activity via blocking the Keap1-Nrf2 interaction and upregulating HO-1 and NQO1. (A) Schematic diagram of the mechanism of action of compound **1** in activating the cellular antioxidative response. (B) Effect of compound **1** on Nrf2 and Keap1 levels in HaCaT cells with or without Keap1 siRNA treatment. The total protein levels of Nrf2 and Keap1 were assessed by Western blotting. (C) Quantification analysis of Nrf2, HO-1, and NQO1 levels in Keap1 knockdown cells. (D) Effects of 1 μM, 3 μM, and 10 μM compound **1**, and 10 μM ML334 on NQO1, HO-1, protein levels in HaCaT cells after 8 h treatment. (E) Quantification analysis of HO-1 and NQO1 levels in HaCaT cells. (F) HaCaT cells were transfected with an ARE-firefly luciferase reporter plasmid, and luciferase activity was measured using the dual-luciferase assay after treatment with compound **1**, compound **2**, itaconate, or ML334. (G) Inducer-stimulated HaCaT cells were treated with 1 μM, 3 μM, and 10 μM compound **1**, and 10 μM ML334 for 8 h. Intracellular ROS levels were detected by DCFH-DA probe and measured using the Flow cytometry. Data are represented as mean ± SD. *p < 0.05, **p < 0.01 vs DMSO group.

Inflammation and oxidative stress are key drivers of ROS, and activated Nrf2 helps to decrease ROS levels within the cell to mitigate the effects of oxidative damage. Hence, we determined the ability of compound **1** to reduce ROS levels in HaCaT cells using ML334 as a control. Compound **1** significantly decreased ROS levels stimulated by the inducer in a dose-dependent manner (1–10 μM), and had stronger antioxidant ability than ML334 at the same concentration (Figure 4G). Overall, compound **1** exerts strong antioxidative effects via Keap1-Nrf2 disruption, thereby upregulating HO-1 and NQO1 and decreasing cellular ROS production.

### Compound **1** Could Upregulate the Level of Skin Barrier Proteins in a 3D RHE Atopic Dermatitis Model

The RHE model is a three-dimensional skin organ culture model in vitro, which simulates the structure and function of human skin.^33^ It is composed of epidermis and dermis, usually constructed by human keratinocytes and fibroblasts, and exhibits the physiological characteristics of human skin. Therefore, we constructed a 3D RHE atopic dermatitis model through the pretreatment of RHE with methyl-β-cyclodextrin (MβCD) and IL-4 (Figure 5A). MβCD damages the skin barrier, while IL-4 triggers a type 2 immune inflammatory response on the damaged skin barrier. Thus, two core pathological features of atopic dermatitis, including barrier dysfunction and immune abnormalities, can be mimicked. In the 3D RHE atopic dermatitis model, we hypothesized that compound **1** could activate the Nrf2 signaling pathway, exert its antioxidant and anti-inflammatory effects, promote the synthesis of key barrier proteins, and improve abnormal skin histopathological features (Figure 5A).

**Figure 5:**
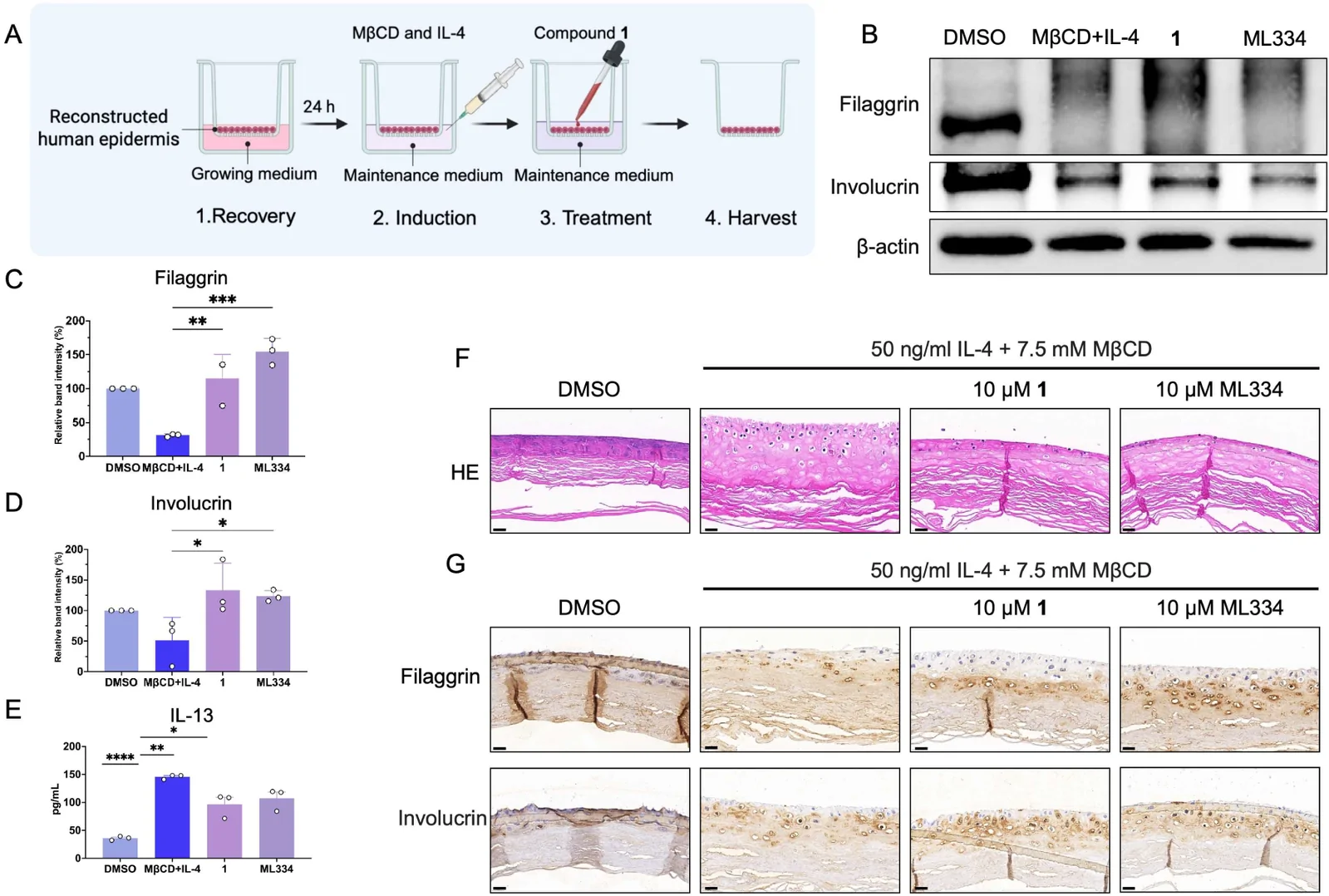
Compound **1** inhibits atopic dermatitis in a 3D RHE model. (A) Schematic diagram of the construction of the 3D RHE atopic dermatitis model. (B) Expression levels of filaggrin and involucrin after treatment with compound **1** (10 μM) and ML334 (10 μM) in 3D RHE model pretreated with MβCD and IL-4, respectively. (C–D) Quantitative analysis of silk protein in the 3D RHE model. MβCD + IL-4: 7.5 mM MβCD treatment for 2 h, then 50 ng/mL IL-4 treatment for 48 h; (E) The relative level of IL-13 in the 3D RHE supernatant determined by ELISA (*n* = 3). (F) H&E staining of 3D RHE after treatment with compound **1** (magnification, ×63). Scale bar = 20 μm. (G) Immunohistochemical analysis of filaggrin and involucrin in the 3D RHE in tissue sections after treatment with compound **1** (magnification, ×63). Scale bar = 20 μm.

We first evaluated the skin penetrability of compound **1** in the 3D RHE model. Compound **1** was topically applied onto the surface of the RHE model, and samples collected at predetermined time points and analyzed by ICP-MS. Compound **1** efficiently penetrated the skin barrier of the RHE model and was distributed into the epidermal and dermal layers, with stable retention in skin tissues within 4 h postadministration, suggesting its suitability for topical delivery (Figure S15). Moreover, compound **1** treatment elevated filaggrin and involucrin levels in atopic dermatitis model cells (Figures 5B–D) and also effectively suppressed the levels of IL-13 induced by MβCD and IL-4 in the 3D RHE supernatant, a cytokine that is significantly upregulated in the atopic dermatitis model (Figure 5E). H&E staining of the treated 3D RHE showed that IL-4/MβCD treatment caused skin barrier damage, while compound **1** treatment significantly alleviated the damage (Figure 5F). Moreover, immunohistochemistry experiments revealed that the compound **1** significantly increased expression of filaggrin and involucrin in treated 3D RHE (Figure 5G). These results indicate that compound **1** can can alleviate skin damage and elevate the expression of skin barrier proteins, filaggrin and involucrin, demonstrating its therapeutic potential for atopic dermatitis

### Compound **1** Alleviated Atopic Dermatitis-Like Symptoms in a DNFB-Induced Atopic Dermatitis-Like Mouse Model

In order to further explore the therapeutic potential of compound **1** *in vivo*, we constructed a mouse model of atopic dermatitis induced by dinitrofluorobenzene (DNFB) to verify the anti-inflammatory activity of compound **1** *in vivo* (Figure 6A). Dexamethasone (DXM), a hormone drug commonly used in clinical atopic dermatitis, was selected as a positive control. After 12 days of consecutive daily topical administration, compound **1** caused milder body weight loss than DXM (Figure 6B). Daily clinical scoring of dorsal skin lesions showed that both compound **1** and DXM groups had a significantly lower AD lesion severity than the DNFB model group (Figure 6C). DNFB-induced mice developed typical atopic dermatitis-like symptoms including erythema, edema, pruritus, and xerosis on the back and ears. Encouragingly, pruritus was notably relieved after 4 days of treatment with compound **1** or DXM (Figure 6D). The spleen and thymus are key immune organs in AD pathogenesis. Less obvious changes in spleen and thymus organ coefficients were observed in the compound **1** group vs the DXM group (Figures 6E and F), demonstrating the preferable biosafety and anti-inflammatory properties of compound **1**.

**Figure 6:**
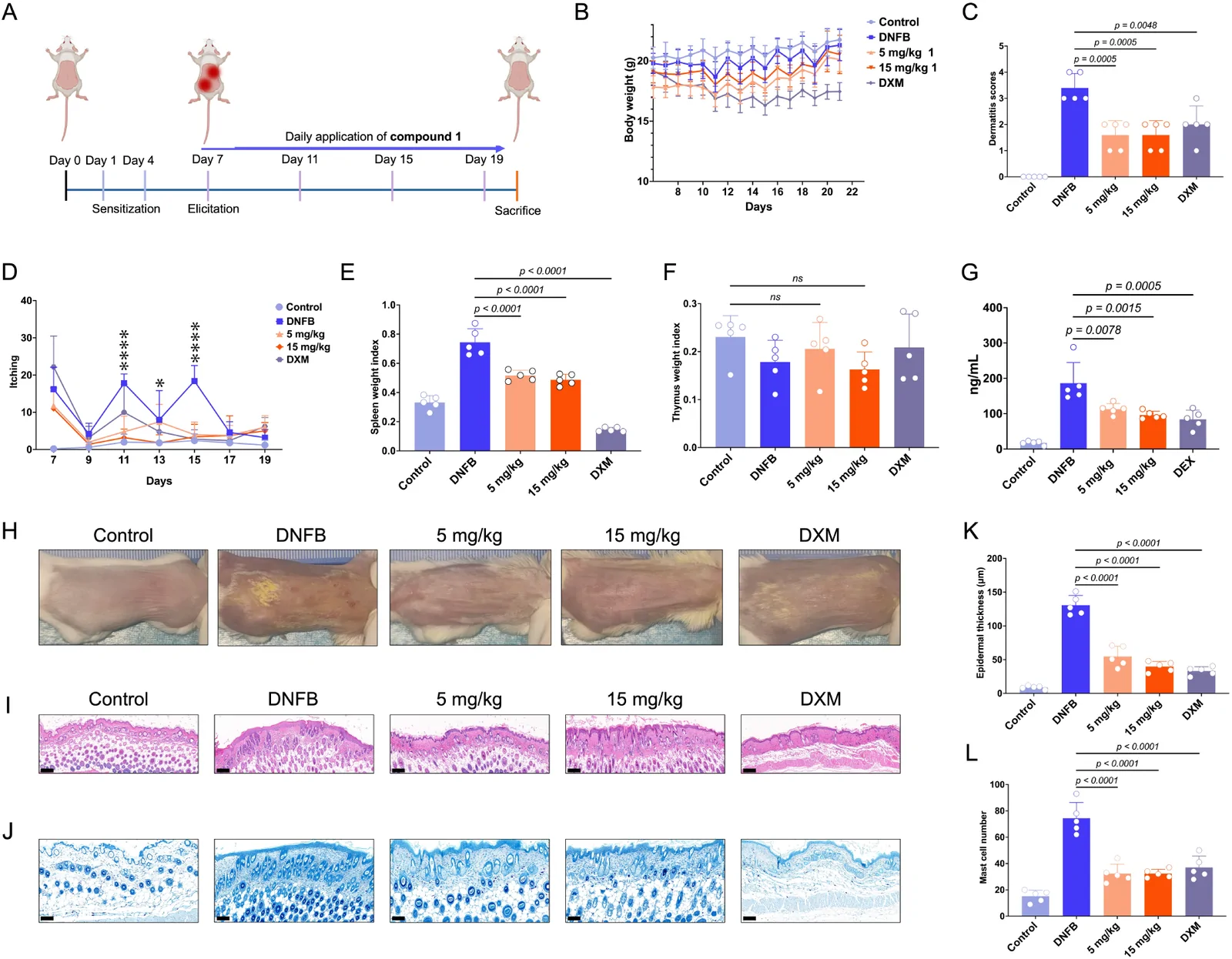
Topical application of compound **1** protects the skin in a DNFB-induced AD-like mouse model. (A) Schematic diagram of the atopic dermatitis-like mice experimentation process. (B) Body weight of animals for therapy. (C) The dermatitis score of the back skin in each group (*n* = 5). (D) Quantification of mice itching times in each group (*n* = 5). (E) The spleen organ index of mice in each group. (F) The thymus organ index of mice in each group. (G) Concentrations of serum IgE through ELISA (*n* = 5). (H) Representative images of back skin lesions in each group. (I) Representative H&E staining images showing the condition of epidermal thickness, edema, and dermal eosinophilic infiltration after compound **1** therapy (scale bar = 200 μm). (J) Representative Toluidine blue staining showing a decrease in the number of mast cell granules in the lesions after compound **1** therapy (scale bar = 100 μm). (K) Quantification of epidermal thickness in the skin lesions of each group. (L) Quantification of mast cells in the skin lesions of each group.

We further detected serum IgE via ELISA to assess compound **1**’s regulation of hypersensitivity. Compound **1** markedly reversed DNFB-induced IgE elevation (Figure 6G). Representative dorsal skin photos confirmed that compound **1** ameliorated DNFB-triggered atopic dermatitis lesions (Figure 6H). DNFB induces skin inflammation featuring epidermal hyperplasia and inflammatory cell infiltration. H&E and toluidine blue staining of dorsal skin tissues showed that compound **1** or DXM greatly restrained inflammatory progression (Figures 6I and J). Quantitative histological analysis verified thinner epidermis and fewer infiltrated mast cells in both treatment groups compared with the DNFB model group (Figures 6K and L).

### The Diagnostic Application of Compound **1** for Discriminating HaCaT from A375 Cells Based on the Level of Keap1 Protein

After studying the therapeutic effect of compound **1** on atopic dermatitis, we turned to the study of the integrated application of compound **1** for evaluating Keap1 protein levels in different cell lines. The emission intensity of compound **1** was elevated in the Keap1 overexpression group, consistent with its ability to bind to Keap1 in cells (Figure S13). The photophysical characteristics of compound **1** were further studied in vitro. We performed a photobleaching assay to compare the photostability of compound **1** with the commercial dye Lyso-Tracker as a benchmark. After 720 s, the mean emission intensity of compound 1 (λ_ex_/λ_em_ = 405/500–750 nm) decreased by 4.6%, whereas the luminescence of Lyso-Tracker (λ_ex_/λ_em_ = 488/500–650 nm) decreased by 20.6% (Figure S14). This result demonstrates the high photostability of compound **1**, indicating it could be employed to visualize Keap1 protein in cells over a long period of time.

Once the relationship between Keap1 protein level and the luminescence of compound **1** was indicated, we next applied the flow cytometry experiments to compare the luminescence intensity of compound **1** in different cell lines with incompatible Keap1 protein levels (Figure 7A). In order to assess the cytotoxicity of compound **1**, A375 skin cancer cells were treated with compound **1** from 0 to 10 μM for 72 h. From the MTT assay, the IC_50_ was calculated to be 9.95 μM (Figure 7B). It should be noted that although compound **1** was moderately cytotoxicity to A375 skin cancer cells, its IC_50_ was over 10 μM in keratinocyte HaCaT cells (Figure 1G), confirming its safety for treating atopic dermatitis, particularly considering its intended topical, rather than systemic delivery. The level of Keap1 protein in HaCaT and A375 cells was measured by Western blotting, which revealed that HaCaT cells contained higher Keap1 levels than A375 cells (Figure 7C). Consistent with this, compound **1** exhibited significantly higher luminescence in the confocal imaging of HaCaT cells compared to A375 cells (Figure 7D). Next, the emission intensity of compound **1** (0–20 μM) in HaCaT and A375 cells was monitored by flow cytometry. The results showed that the luminescence signal observed in the cells increased with the concentration of compound **1** (Figure 7E). Quantitation of the flow cytometry data confirmed that the emission intensity of compound **1** was correlated with the level of Keap1 protein (Figure 7F), demonstrating its capability to detect and visualize Keap1 protein levels in living cells and the potential in the diagnosis of atopic dermatitis.

**Figure 7:**
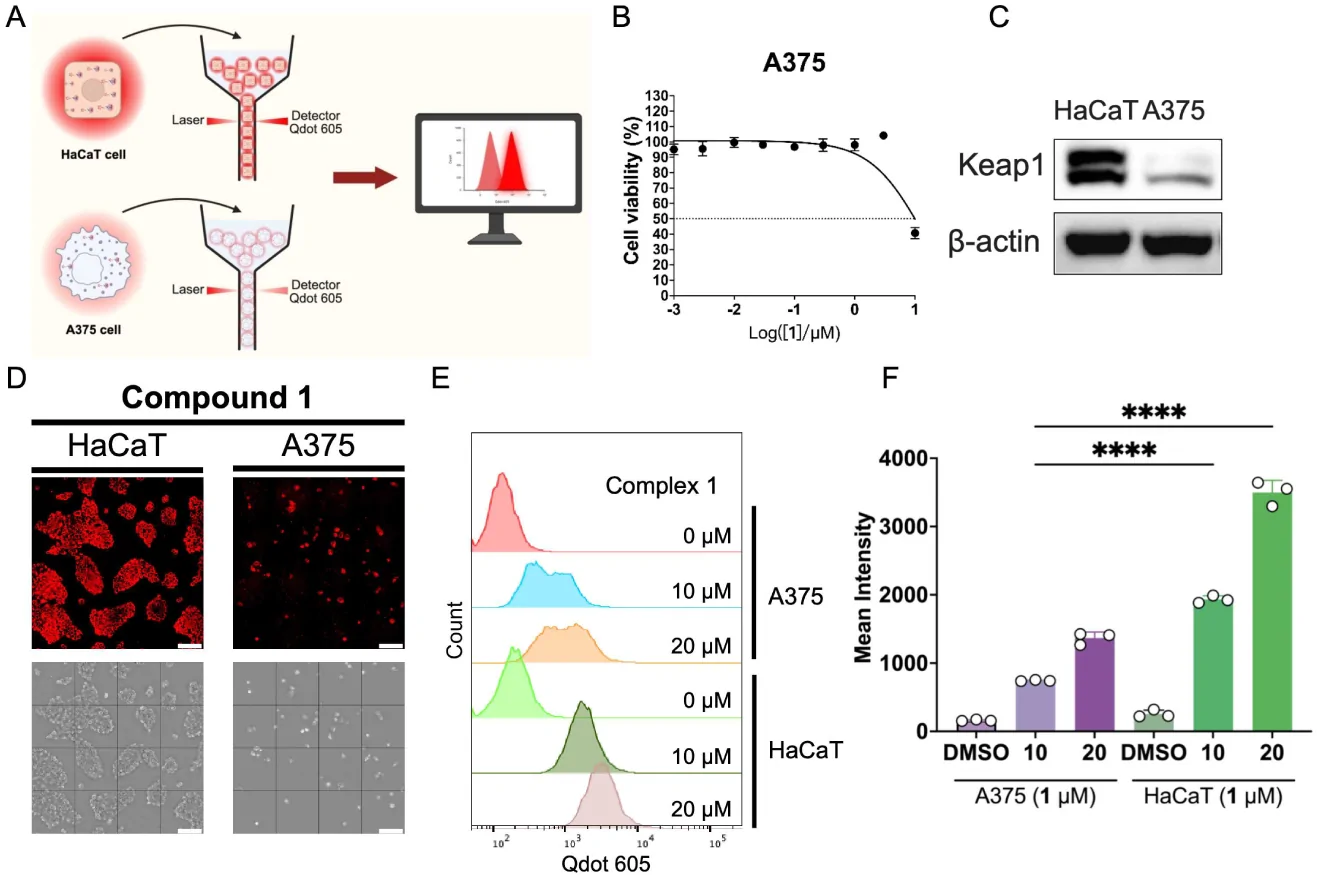
Application of compound **1** in the integration of detection and treatment of atopic dermatitis. (A) Schematic diagram of the application of compound **1** in different cell lines. (B) The cytotoxicity of compound **1** against A375 cells for 72 h. Cells were treated with 0–10 μM of compound **1** for 72 h, and cytotoxicity was detected using the MTT assay. (C) Western blot assay for Keap1 protein in HaCaT and A375 cell lines. (D) HaCaT and A375 cells preincubated with 10 μM compound **1** for 2 h. Cell images were recorded at λ_ex_/λ_em_ = 405/600–720 nm. Cells were washed three times with PBS and then imaged using an HCS confocal microscope. Scale bar = 100 μm. (E) Flow cytometry assay to evaluate the luminescence intensity of compound **1** in HaCaT and A375 cell lines. (F) Quantification analysis of luminescence intensity of compound **1** in the flow cytometry assay.

## Discussion and Conclusions

Atopic dermatitis is a common chronic recurrent inflammatory skin disorder characterized by skin barrier dysfunction and elevated IL-4/IL-13 responses. Current targeted drug discovery strategies mainly concern the JAK/STAT pathway or directly inhibit the IL-4 receptor. Another potential therapeutic target is the transcription factor Nrf2 due to its role in regulating redox balance, suppressing inflammation, and enhancing skin barrier protein expression. The endogenous metabolite itaconate and its derivative were previously identified as potential Keap1-Nrf2 PPI inhibitors through alkylating the Keap1 protein.^18^ However, their activities are too low to treat atopic dermatitis, while traditional medicinal chemistry efforts to improve the binding of these inhibitors have been slow. Here, we propose a concept of the incorporation of itaconate into a luminescent iridium­(III) complex for improving its Keap1-Nrf2 PPI blocking activity through confining itaconate to the cytoplasm and while increasing its PPI inhibiting through the steric bulk of iridium­(III) complexes. The concept of the design indicates the important determinants for Keap1-Nrf2 inhibitory activity, which are (1) having the electrophilic α,β-unsaturated carboxylic acid on the *N^N* ligand for targeting Keap1, (2) a linker to retain the activity of itaconate, (3) the dfpq *C^N* ligand for enabling NIR emission,^26,28^ (4) subcellular targetability of iridium­(III) complexes confines itaconate to the cytoplasm, and (5) the octahedral geometry of the iridium­(III) complex provides steric bulk for disrupting the Keap1-Nrf2 PPI.

The iridium­(III)-itaconate conjugate **1** exhibited direct interaction with Keap1, as revealed by a CETSA experiment in HaCaT cell lysates, while ITC determined a dissociation constant of *K*_d_ = 0.627 ± 0.282 μM. Moreover, FP and co-IP experiments showed that compound **1** inhibited the Keap1-Nrf2 PPI with an EC_50_ of 14.2 μM, which was superior to the positive control ML334. The results of colocalization imaging and ICP-MS experiments confirmed that compound **1** specifically localizes in the cytoplasm where the Keap1-Nrf2 PPI mainly exists. These are consistent with our hypothesis that conjugation of an iridium­(III) complex not only confines itaconate into the cytoplasm but also improves its PPI inhibitory activity through the steric bulk of the octahedral iridium­(III) complex, thus synergistically amplifying the Keap1-Nrf2 PPI inhibition ability of itaconate. This is mechanically different from traditional PPI inhibitors, which are typically developed by iterative structural optimization.

ROS is highly associated with atopic dermatitis, while antioxidant proteins can reduce ROS and decrease the inflammatory burden. Further mechanistic studies demonstrated that compound **1** could upregulate downstream key antioxidant proteins (HO-1 and NQO1) while decreasing cellular ROS levels by enhancing Nrf2 in HaCaT cells. Moreover, compound **1** showed stronger activity than ML334 under the same conditions, further verifying its strong PPI-disruption ability. Furthermore, the therapeutic potential of compound **1** was confirmed in an RHE 3D skin model, where it inhibited IL-13 expression and promoted filaggrin/involucrin protein levels, demonstrating its potential in treating atopic dermatitis. This is the first example of conjugating itaconate to an iridium­(III) complex for confining its cellular localization and sterically enhancing PPI inhibitory activity, making compound **1** a suitable candidate for treating atopic dermatitis. The amelioration of atopic dermatitis-like symptoms by compound **1**, as indicated by downregulated serum IgE levels, decreased epidermal hyperplasia, and reduced mast cell infiltration, and with minimal adverse effects on key immune organs, highlights its potential as an antiatopic dermatitis compound.

Owing to the inherent optical properties of the iridium­(III) center, compound **1** shows a maximal luminescence emission at 710 nm, offering NIR emission that is less susceptible to biological interference. It should be noted that most reported Keap1-Nrf2 PPI inhibitors, including itaconate and ML334, are nonfluorescent or weakly fluorescent. These inhibitors would require fluorescent tagging to visualize their subcellular locations, which introduces additional complexity and may also affect their biological effects. In contrast, the intrinsic luminescence of compound **1** allows it to be used for subcellular imaging in HaCaT cells and for discriminating HaCaT cells from A375 cells through both cell imaging or flow cytometry based on the expression level of Keap1. This property also highlights the potential use of compound **1** in dissecting the biological roles of Keap1-Nrf2 PPI in atopic dermatitis as well as serving as a contrast imaging agent to accurately diagnose atopic dermatitis. In summary, compound **1** is a promising theranostic complex for atopic dermatitis therapy through its ability to engage and image Keap1.

In this work, we conjugated itaconate to an NIR iridium­(III) complex to develop an effective inhibitor of subcellular Keap1-Nrf2 PPI for treating atopic dermatitis. Compound **1** selectively accumulated in the cytoplasm and bound to Keap1 through its electrophilic α,β-unsaturated carboxylic acid moiety, restoring Nrf2 activity and inducing Nrf2 translocation into the nucleus for anti-inflammatory activity. Cellular mechanism experiments showed that compound **1** stabilizes Keap1 protein, promotes Nrf2 nuclear translocation, and activates ARE activity in HaCaT cells. Consequently, this complex can upregulate anti-inflammatory HO-1 and NQO1 protein levels and decrease ROS levels to protect skin cells. Further experiments showed that compound **1** can decrease IL-13 production to block their effects and induce the expression of skin barrier proteins filaggrin and involucrin in an atopic dermatitis model. Compound **1** also reversed skin inflammation and immune hyperactivation while avoiding the severe toxicities associated with hormone therapies, highlighting its great potential as a safe and effective candidate for managing atopic dermatitis. Furthermore, compound **1** could discriminate HaCaT cells from cancer cells, supporting its potential clinical application as a contrast imaging agent to diagnose atopic dermatitis. We believe that this complex could serve as a good starting point for the development of luminescent metallodrugs to break PPI for atopic dermatitis theranostics.

## Experimental Section

### Chemicals

2-(2-Pyridyl)­benzimidazole, ethyl 4-bromobutyrate, and *N*-(3-bromopropyl)*tert*-butyl carbamate were purchased from Shanghai Haohong Technology Co., Ltd. (Shanghai, China). Itaconic anhydride was purchased from Bide Pharmaceutical Technology Co., Ltd. (Shanghai, China). Iridium chloride hydrate (IrCl_3_·*x*H_2_O) and other reagents, unless otherwise specified, were purchased from J&K Chemical Ltd. (Beijing, China) and were used on receipt.

### Synthesis of Compound **S1**

A mixture of 2-(2-pyridyl)­benzimidazole (8.0 mmol) and KOH (16 mmol) in acetone (40 mL) was stirred at 60 °C for 6 h. Then added 3-(boc-amino)­propyl bromide (12 mmol) to the mixture, which was stirred at 60 °C overnight. Then, the reaction mixture was cooled down and directly purified by silica column chromatography to afford the desired product. Yield: 70%. ^1^H NMR (500 MHz, CDCl_3_-d) δ 8.76 (d, *J* = 4.9 Hz, 1H), 8.45 (d, *J* = 8.0 Hz, ^1^H), 7.95–7.77 (m, 2H), 7.56–7.17 (m, 4H), 6.06 (d, *J* = 6.3 Hz, 1H), 4.86 (t, *J* = 7.0 Hz, 2H), 3.19 (t, *J* = 6.3 Hz, 2H), 2.19 (q, *J* = 6.7 Hz, 2H), 1.48 (s, 9H). ^13^C NMR (126 MHz, CDCl_3_-d) δ 156.09, 150.28, 149.69, 148.40, 142.62, 137.26, 136.34, 125.38, 124.01, 123.61, 122.87, 120.24, 110.37, 79.04, 42.90, 37.12, 29.79, 28.49. ESI-MS: Calcd for C_20_H_24_O_2_N_4_ [M + H]^+^ 353.19. Found: 353.30.

### Synthesis of Compound **S2**

Compound **S1** was dissolved into a 4 N HCl dioxane solution, and the mixture was stirred for 1 h and evaporated to obtain compound **S2**. The product was directly used in the next step without further purification.

### Synthesis of Compound **S3**

Monobutyl itaconate (1.2 mmol) was dissolved in 15 mL of DCM, then EDCI (2.0 mmol) and HOBt (2.0 mmol). Finally, Compound **S2** (1.0 mmol) and DIPEA (5.0 mmol) were added into the mixture. The mixture was stirred at rt overnight. The mixture was purified by silica column chromatography to afford the desired product **S3**. Yield: 66%. ^1^H NMR (500 MHz, CDCl_3_-d) δ 8.73 (dt, *J* = 19.8, 6.9 Hz, 2H), 8.51 (q, *J* = 5.1 Hz, 1H), 8.21 (d, *J* = 8.5 Hz, 1H), 7.80–7.66 (m, 2H), 7.14 (q, *J* = 5.5, 5.0 Hz, 1H), 3.59 (q, *J* = 5.8 Hz, 2H), 3.49–3.33 (m, 2H), 2.43 (m, 3H), 1.41 (s, 9H). ^13^C NMR (126 MHz, CDCl_3_) δ 166.01, 157.50, 157.16, 155.18, 149.92, 149.03, 148.23, 142.37, 125.06, 122.06, 121.38, 118.05, 80.07, 42.14, 40.01, 28.35, 21.20. ESI-MS: Calcd for C_20_H_20_N_4_O_3_: 364.15. Found: 363.24.

### Synthesis of Compound **S4**

A mixture of compound **S3** (5.0 mmol) and LiOH (25.0 mmol) was used in MeOH (10 mL). H_2_O (3 mL) was then added, and the mixture was stirred overnight at rt. Afterward, the organic solvent was evaporated under reduced pressure. The residue was rinsed with water and DCM, and an aqueous layer was collected. The pH of the solution was adjusted to 7, and then, it was filtered to obtain the product **S4** for direct use without further purification.

### Synthetic Procedure of Compound **1**

IrCl_3_·*x*H_2_O reacted with 2.1 equiv of cyclometalated ligand dfpq at 130 °C in 2-methoxyethanol: H_2_O (v/v, 3:1) under N_2_ overnight. After cooling to room temperature, the mixture was filtered and washed with deionized water three times and ether three times to yield the cyclometalated dichloro-bridged dimer (Ir_2_(dfpq)_4_Cl_2_) without further purification. A suspension of Ir_2_(dfpq)_4_Cl_2_ (0.08 mmol) and compound **S4** (0.16 mmol) in a mixture of DCM:MeOH (1:1, 6 mL) was stirred under rt overnight. The reaction mixture was added with excess solid ammonium hexafluorophosphate (NH_4_PF_6_), and then stirred for another 0.5 h. The reaction mixture was purified by column chromatography to afford final probe **1**. Yield: 48%. ^1^H NMR (500 MHz, DMSO-*d*_6_) δ 8.55 (d, *J* = 8.4 Hz, 1H), 8.37–8.17 (m, 4H), 8.16–8.06 (m, 1H), 8.02–7.76 (m, 5H), 7.51 (t, *J* = 7.9 Hz, 1H), 7.30 (td, *J* = 7.8, 4.9 Hz, 3H), 6.93 (dt, *J* = 22.1, 7.4 Hz, 2H), 6.83– 6.71 (m, 2H), 6.66 (d, *J* = 7.8 Hz, 1H), 6.10 (d, *J* = 1.8 Hz, 1H), 5.74 (dd, *J* = 6.3, 3.0 Hz, 5H), 4.69–4.44 (m, 2H), 3.39 (d, *J* = 5.6 Hz, 3H), 3.19–3.12 (m, 1H), 3.07 (s, 1H), 2.88 (d, *J* = 3.9 Hz, 4H), 2.52 (d, *J* = 3.6 Hz, 2H). ^13^C NMR (101 MHz, DMSO-*d*_6_) δ 170.0, 168.2, 165.6, 164.8, 154.3, 154.2, 153.2, 152.6, 152.5, 151.8, 149.6, 148.9, 146.4, 145.8, 144.5, 141.5, 137.6, 137.5, 137.4, 136.5, 136.4, 136.0, 135.4, 135.2, 132.0, 131.6, 131.1, 129.5, 127.7, 126.7, 126.4, 125.7, 123.6, 116.7, 115.8, 113.6, 113.1, 110.9, 110.7, 43.4, 35.8, 29.5, 27.6, 27.1. ESI-MS: Calcd for C_50_H_38_F_4_IrN_8_O_3_ [M–PF_6_]^+^: 1067.2632. Found: 1067.2526. Purity: >95% (HPLC).

Compound **2**: Reported.^28^

### Cellular Thermal Shift Assay (CETSA)

HaCaT cell lysates were incubated with compound **1** (20 μM) or DMSO at room temperature for 0.5 h. The lysates were divided and heated individually at 45 °C, 50 °C, 55 °C, 60 °C, 65 °C, 70 °C, and 75 °C. The supernatants of the heated lysates were collected after centrifugation and then analyzed by WB with the Keap1, Nrf2, and β-actin antibodies.

### Isothermal Titration Calorimetry (ITC)

The ITC experiments were performed on a MicroCal PEAQ-ITC microcalorimeter (Malvern Panalytical GmbH, Germany). Experiments were carried out at 25 °C, and 150 μM compound **1** was titrated in 2 μL steps into a 5 μM Keap1 solution for 19 times in the calorimeter cell. The instrument software (MicroCal PEAQ-ITC Analysis) was used for the result readout.

### Western Blot (WB) and Coimmunoprecipitation (Co-IP) Assay

HaCaT cells were seeded at a density of 2 × 10^6^ cells in a 6-well plate. Cell lysates were harvested after incubation with compound **1** or ML334 for 8 h. A 10% SDS-PAGE gel was used to separate the cell lysates, then transferred to a PVDF membrane (Bio-Rad, USA). The membranes were incubated with antibodies anti-Keap1 (1:1000), Nrf2 (1:1000), HO-1 (1:1000), NQO1 (1:1000), and β-actin (1:1000) overnight at 4 °C. The membranes were washed with wash buffer three times. After 2 h incubation of secondary antibody (1:1000) at room temperature, protein blots were detected using enhanced chemiluminescent Plus reagents (GE Healthcare) and analyzed by Image Lab. Co-IP between Keap1 and Nrf2 was performed following the protocol from Life Technologies. Briefly, lysates from 2 × 10^6^ HaCaT cells were incubated with anti-Keap1 or Rabbit mAb IgG (1:1000, Abcam, USA; Cat#: ab191866) overnight. Then, samples were incubated with 20 μL speedbeads (Cytiva, Sera-Mag SpeedBeads Protein A/G Magnetic Particles; Cat#: 17152104010150) for 4 h. The speedbeads were washed and analyzed by Western blot with the Nrf2 antibody.

### H&E Staining of RHE

The 3D RHE were bought from Episkin (France). 3D RHE model morphology and barrier proteins were changed after treatment with methyl-β-cyclodextrin (MβCD). The cells were pretreated with 7.5 mM MβCD for 2 h and then cultured with 50 ng/mL IL-4 for 48 h. After treatment with 100 μL of 10 μM compound **1** or ML334, the 3D RHE models were fixed, dehydrated, waxed, transparentized, embedded, and sectioned. Hematoxylin and eosin were used for staining. Finally, the morphology of the 3D RHE model was observed using an optical microscope.

### Construction of Atopic Dermatitis-Like Mouse Model Induced by Dinitrofluorobenzene (DNFB)

Female Balb/c mice aged 6–8 weeks old were housed at 25 ± 2 °C under a 12 h light/dark cycle with free access to food and water. Forty-eight mice were randomly assigned to six groups: normal control group, DNFB model group, low-dose compound **1** group (5 mg/kg), high-dose compound **1** group (15 mg/kg), and positive control group (dexamethasone, 3 mg/kg). A 2.5 × 4 cm area of dorsal hair was shaved on day 0. On days 1 and 4, all groups except the normal control were sensitized by the topical application of 50 μL 0.5% DNFB solution (acetone/olive oil = 3:1, v/v) onto the shaved skin. On days 7, 11, 15, 19, and 21, DNFB-challenged groups received 100 μL 0.2% DNFB solution for rechallenge in the same manner, while the control group was treated with an equal volume of acetone solvent.

Starting from day 7, compound **1** or dexamethasone was topically administered once daily for 14 consecutive days; the normal control group received normal saline topically. Skin lesions on the dorsum were observed and recorded from days 7 to 21. Blood samples were collected from the ophthalmic venous plexus and centrifuged to isolate the serum. The clinical skin severity was scored daily from 0 to 3 based on four indicators: erythema, edema, scaling/erosion, and dryness. Detailed AD lesion severity was scored daily from 0 to 3 across four indicators: erythema/hemorrhage, edema, peeling/erosion, and dryness.

For erythema/hemorrhage: 0 = none; 1 = faint punctate erythema; 2 = mottled red papules; and 3 = dark red irregular bumps.

For edema: 0 = none; 1 = mild swelling; 2 = local edema with spot exudate; 3 = extensive edema, heavy exudate, and crusts.

For peeling/erosion: 0 = intact skin; 1 = mild erosion; 2 = moderate erosion; and 3 = severe infected erosion.

For dryness: 0 = normal skin; 1 = slight dryness; 2 = moderate dryness with scaling; and 3 = severe dryness with heavy peeling.

All animal experiments were performed following the protocols evaluated and approved by Zhuhai Bestest Bio-Tech Co., Ltd. (Ethics Approval Number: IAC26W004(14)).

## Supplementary Material





## Data Availability

The data supporting this article have been included as part of the (Supporting Information SI). Additional information: experimental details, synthetic steps of the compound, spectral data of the compound, and Western blot whole membrane data.

## Notes

^1^H NMR and ^13^C NMR for all compounds; HRMS and HPLC spectra for compound **1**; synthetic routes for compounds; detailed experimental procedures for luminescence experiments; additional experimental details, materials, and methods for biological evaluations and animal experiments, including photographs of experimental results (DOCX)
Molecular formula strings (CSV)

## Abbreviations

BSA: bovine serum albumin
CETSA: cellular thermal shift assay
EDCI: 1-ethyl-3-(3-dimethylaminopropyl)­carbodiimide hydrochloride
ESI-MS: electrospray ionization mass spectrometry
HPLC: high-performance liquid chromatography
HRMS: high-resolution mass spectrometry
ICP-MS: inductively coupled plasma mass spectrometry
IL: interleukin
IL-4R: IL-4 receptor
ITC: isothermal titration calorimetry
Keap1: Kelch-like ECH-associated protein
KOH: potassium hydroxide
LC-MS: liquid chromatography mass spectrometry
MβCD: methyl-β-cyclodextrin
NQO1: NAD­(P)H dehydrogenase [quinone] 1
MLCT: metal–ligand charge transfer
Nrf2: Nuclear factor erythroid 2-related factor 2
PPI: protein–protein interaction
RHE: reconstructed human epidermis
ROS: reactive oxygen species
