# Supplementary material for: A Subcellular Keap1-Nrf2 Disruption Strategy for Atopic Dermatitis Therapy
Source: J Med Chem. 2026 Aug 21;69(17):20326–37. doi: 10.1021/acs.jmedchem.6c00772 (PMC13576393; doi:10.1021/acs.jmedchem.6c00772)
Supplement: Supplementary file 1 [file jm6c00772_si_001.docx]

**Supporting Information**

**A Subcellular Keap1–Nrf2 Disruption Strategy for Atopic Dermatitis Therapy**

Zixi Zhang^a,d,1^, Jia Wu^b,c,1^, Jingqi Liu^a^, Daniel Shiu-Hin Chan^e^, Chun-Yuen Wong^e^, Jing Wang^a,d,*^, Chung-Hang Leung^b,c*^, Wanhe Wang^a,d,f*^

^a^ School of Life Science and Technology, Northwestern Polytechnical University, 1 Dongxiang Road, Xi'an, Shaanxi, 710072, China

^b^ State Key Laboratory of Mechanism and Quality of Chinese Medicine, University of Macau, Taipa, 999078, Macao, China.

^c^ Institute of Chinese Medical Sciences, University of Macau, Macao SAR, China

^d^ Research & Development Institute of Northwestern Polytechnical University in Shenzhen, 45 South Gaoxin Road, Shenzhen, Guangdong, 518057, China

^e^ Department of Chemistry, City University of Hong Kong, 999077, Hong Kong, China

^f^ College of Chemistry and Materials Science, State Key Laboratory of Bioactive Molecules and Druggability Assessment, Jinan University, Guangzhou, 510632, China

*Email: whwang0206@nwpu.edu.cn (Wanhe Wang); duncanleung@um.edu.mo (Chung-Hang Leung); jwang0321@nwpu.edu.cn (Jing Wang)

^1^These authors are equally contributed.

**Table of Contents**

**[Part A: Synthesis experiment	S4](#_Toc531236598)**

**[Scheme S1	S4](#_Toc1403982007)**

**[Scheme S2	S4](#_Toc175278413)**

**[Figure S1	S5](#_Toc1704207254)**

**[Figure S2	S5](#_Toc1621917939)**

**[Figure S3	S6](#_Toc1564869402)**

**[Figure S4	S6](#_Toc527814605)**

**[Figure S5	S7](#_Toc1872604125)**

**[Figure S6	S7](#_Toc1484682090)**

**[Figure S7	S8](#_Toc1439392137)**

**[The HPLC analysis of compound 1	S8](#_Toc460363104)**

**[Figure S8	S8](#_Toc2086592434)**

**[Part B: Luminescence experiments	S9](#_Toc951082728)**

**[Photophysical measurement	S9](#_Toc1126624875)**

**[Lifetime measurement	S9](#_Toc820958526)**

**[Part C: Biological experiments	S9](#_Toc267514507)**

**[Materials and cell culture	S9](#_Toc1433045978)**

**[MTT assay	S9](#_Toc1174651141)**

**[Expression and purification of Keap1 and Nrf2 proteins	S10](#_Toc544559916)**

**[Itaconate competition Assay	S10](#_Toc1990688345)**

**[Keap1 expression knockdown	S10](#_Toc1851277802)**

**[Colocalization assay	S10](#_Toc1682940478)**

**[Fluorescence polarization assay	S10](#_Toc673499109)**

**[Immunofluorescence staining	S11](#_Toc113221626)**

**[Dual luciferase reporter gene assay	S11](#_Toc245356940)**

**[Detection of ROS	S11](#_Toc545488340)**

**[Luminescence intensity of compound 1	S11](#_Toc414841337)**

**[Enzyme-linked immunosorbent assay (ELISA)	S11](#_Toc1931304696)**

**[Statistical analysis	S11](#_Toc222701267)**

**[Table S1.	S11](#_Toc2023681395)**

**[Figure S9.	S12](#_Toc167204579)**

**[Figure S10.	S12](#_Toc1298748977)**

**[Figure S11.	S13](#_Toc1050268331)**

**[Figure S12.	S14](#_Toc1691744424)**

**[Figure S13.	S14](#_Toc465047888)**

**[Figure S14.	S14](#_Toc1366862183)**

**[Figure S15	S15](#_Toc1220137722)**

**[Figure S16	S15](#_Toc533348451)**

# **Part A: Synthesis experiment**

**Scheme S1**. Synthesis of compound **1**. Reagents and conditions: a) KOH, acetone, 60 °C, overnight; b) HCl in dioxane, overnight; c) Monobutyl itaconate, DCM, N_2_, overnight; d) LiOH, MeOH/H_2_O (10/3), rt, overnight e) Ir_2_(dfpq)_4_Cl_2_ DCM/MeOH, rt, overnight; then NH_4_PF_6_ for 0.5 h.

‘

Scheme S2**.** Synthesis of control compound **2**. Reagents and conditions: a) KOH, acetone, 60 °C, overnight; b) Ir_2_(dfpq)_4_Cl_2_ DCM/MeOH, rt, overnight; then NH_4_PF_6_ for 0.5 h.

**Figure S1.** ^1^H NMR spectrum of compound **S3**.

**Figure S2.** ^1^C NMR spectrum of compound **S3**.

**Figure S3**. ^1^H NMR spectrum of compound **S4**.

**Figure S4**. ^13^C NMR spectrum of compound **S4**.

**Figure S5**. ^1^H NMR spectrum of compound **1**.

**Figure S6**. ^13^C NMR spectrum of compound **1**.

**Figure S7**. HRMS spectrum of compound **1**.

## **The HPLC analysis of compound 1**

The purity of compound **1** was examined by Alliance HPLC system e2695 using an Agilent Extend-C18 column (4.6 mm × 250 mm, 5 μm). The samples were prepared with a final concentration of 1 mg/mL in ACN, and the volume injected was 5 μL with a flow rate of 1.0 mL/min. Mobile phase A is Milli-Q H_2_O (with 0.1% mL/min trifluoroacetic acid (TFA)). And mobile phase B is ACN. The mobile phase gradient was initially set for 5% ACN, 5–95% ACN from 0 min to 25 min, ended up as 95–5% ACN from 25 min to 30 min. UV absorbance was monitored at 254 nm.

**Figure S8.** The HPLC chromatographic spectrum of compound **1**.

# **Part B: Luminescence experiments**

### **Photophysical measurement**

Luminescence emission spectra were recorded on Hitachi F-4700 luminescence spectrophotometer at ambient temperature. The excitation slit and emission slit were both set at 5.0 nm, PMT voltage was set at 500 V, with the scan speed of 1200 nm/min. UV-vis absorption spectra were recorded on a U-3900 Spectrophotometer (Hitachi, Japan).

### **Lifetime measurement**

Lifetime were determined with a Multi-Channel Scaling (MCS) mode on the FLS1000 Photoluminescence Spectrometer equipped with a VPL-365 diode laser. Under excitation at 365 nm, the decay curve of the compound (10 μM) in ACN was recorded with a delay course of 5 μs, while LUDOX® HS-40 colloidal silica was used as a scattering solution. The lifetime value of compounds was obtained by fitting curves of the decay curves on FLS1000 Photoluminescence Spectrometer.

# **Part C: Biological experiments**

### **Materials and cell culture**

Compound **1** was dissolved completely in DMSO. Methyl-β-cyclodextrin and ML334 were purchased from MedChemExpress (Houston, CA, USA). 3,4,5-Trimethoxybenzaldehyde was acquired from Macklin Biochemical Co., Ltd (Shanghai, China). 3-(4,5-dimethylthiazol-2-yl)-2,5-diphenyltetrazolium bromide (MTT) was purchased from Sigma-Aldrich (St. Louis, Missouri, USA). Lipo3000 and human IL-4 recombinant protein were obtained from Thermo Fisher Scientific (Waltham, MA, USA). The antibodies were purchased as follows: anti-Keap1 (Cell Signaling Technology, Danvers, MA, USA; Cat# 8047S), anti-Nrf2 (Proteintech, Wuhan, China; Cat# 16396-1-AP), anti-HO-1 (Cell Signaling Technology, Danvers, MA, USA; Cat# 5853S), anti-NQO1 (Cell Signaling Technology, Danvers, MA, USA; Cat# 3187S), and anti-β-actin (Absin, Shanghai, China; Cat#: abs137975). The reconstructed human epidermises were bought from Episkin (France). The human keratinocyte cell line HaCaT and human normal liver cell line LO2 were procured from Cellverse (Shanghai, China), and the human embryonic kidney cell line HEK293T and human umbilical vein endothelial cell line HUVEC were purchased from American Type Culture Collection (Manassas, VA, USA). Dulbecco’s modified Eagle’s medium (DMEM), penicillin and streptomycin were purchased from Gibco BRL (Gaithersburg, MD, USA). Cells were cultured in DMEM with 10 % FBS, 100 units/mL penicillin, 100 μg/mL streptomycin. Fetal bovine serum (FBS).

### **MTT assay**

HaCaT, LO2, HUVEC and HEK293T cell lines were seeded at 1 × 10^4^ cells per well in 96-well plates and incubated overnight at 37 °C in a humidified CO_2_ incubator to ensure attachment. The cells were then treated by different concentrations of compound **1** for 72 h at 37 °C. The final concentration of DMSO was 0.1% or lower in all cases (including controls). Afterwards, the medium was replaced with 25 μL of 3-(4,5-dimethylthiazol-2-yl)-2,5-tetrazolium bromide (MTT) reagent (1 mg/mL). After 4 h incubation, the medium was replaced with 100 μL of DMSO and the absorbance values were measured using a microplate reader at 570 nm.

### **Expression and purification of Keap1 and Nrf2 proteins**

The plasmid pET28a-His6-Keap1 was purchased from Addgene and extracted using Plasmid Mini Preparation Kit (Beyotime, D0007M) according to the manufacturer’s instruction. The Keap1 plasmid was then transformed into the expression strain *E. coli* (BL21) (DE3). Expanding *E. coli* (BL21) (DE3) culture in lysogeny broth medium with 50 μg/mL kanamycin at 37 °C until the OD600 reached 0.5–0.8. BL21 was induced with 100 μM isopropyl-β-d-thiogalactopyranoside (IPTG) at 18 °C for 16 h and harvested by centrifugation at 8000g for 5 min, following with homogenized by sonication in freeze Phosphate Buffered Saline buffer. Then the precleared lysates were applied to His GraviTrap columns (GE Healthcare), washing by the elution buffer (50 mM Tris-HCl, 150 mM NaCl, 150 mM imidazole, 10% glycerol, pH 7.8-8.0) and purifying with the buffer (20 mM Tris-HCl, 50 mM NaCl, 2 mM dithiothreitol (DTT), 10% glycerol, pH 7.8-8.0) . The Keap1 protein was imaged and quantified by SDS-PAGE by staining with Coomassie brilliant blue R250.

### **Itaconate competition Assay**

HaCaT cells were seeded in glass-bottomed confocal dish (SPL Life Science, Kyong-Gi, South Korea). After 24 h incubation, cells were treated with 1 μM or 3 μM of free itaconate for 4 h prior followed by treating with compound **1** at 10 μM. The cells were washed with PBS, and images were recorded by confocal fluorescence microscopy with excitation at 405 nm after 2 h incubation.

### **Keap1 expression knockdown**

The sequences of Keap1 siRNA are 5'-GGCCUUUGGCAUCAUGAACTT-3' (sense) and 5'-GUUCAUGAUGCCAAAGGCCTG-3' (antisense). The sequences of control siRNA are 5'-CCAUAAUCGUCCUCACCAATT (sense) and 5'-UUGGUGAGGACGAUUAUGGTG-3' (antisense). Keap1 siRNA and control siRNA were transfected with Lipo3000 reagent (Thermo Fisher Scientific) into 1 × 10^6^ HaCaT cells following the manufacturer’s instruction. After incubation for 48 h, Keap1 protein expression and functional experiments were performed.

### **Colocalization assay**

HaCaT cells were spread in a glass-bottomed confocal dish (SPL Life Science, Kyong-Gi, South Korea). The cells were treated with compound **1** for 2h. Cells were washed again with PBS (3×) then imaged using a Leica TCS SP8 confocal laser scanning microscope. After the first cell image, cells were incubated in Phosphate Buffered Saline (PBS) containing Hoechst 33258 for 30 min and then imaged using a Leica TCS SP8 confocal laser scanning microscope.

### **Fluorescence polarization assay**

Fluorescence polarization (FP) assay was performed according to the manufacturer’s instruction (BPS Bioscience, San Diego, CA). The tested compounds were dissolved in the provided buffer at the indicated concentrations. Then, the required volumes of Nrf2 peptide, Bovine Serum Albumin (BSA), Keap1 protein and assay buffer were added to each well followed by incubation for 0.5 h, and the fluorescent polarization value was recorded.

### **Immunofluorescence staining**

HaCaT cells were plated on 35 mm confocal dishes and treatment with compound **1** for 1 h or 2 h. The cells were fixated with 4% paraformaldehyde for 0.5 h and then permeabilized with 0.5% Triton X-100 for 0.25 h. After that, cells were covered with 5% bovine serum albumin for 0.5 h at room temperature followed by anti-Nrf2 antibody (Proteintech, Wuhan, China; Cat# 16396-1-AP) incubation and the goat anti-rabbit IgG FITC tagged secondary antibody (A0562, Beyotime) incubation. Images were acquired on a Leica TCS SP8 confocal laser scanning microscope.

### **Dual luciferase reporter gene assay**

HaCaT cells were seeded in a six-well plate and incubated for 24 h until 80% confluence. The ARE-luciferase vector or a control plasmid with TurboFect transfection reagent (Thermo Fisher Scientific) were transfected into HaCaT cells according to the manufacturer’s instruction. After incubation for 48 h, HaCaT cells were then treated with compound **1** (10 µM), ML334 (10 µM) or DMSO for 8 h. The ARE activity was finally detected as instructed (Beyotime Dual Luciferase Reporter Gene Assay Kit).

### **Detection of ROS**

HaCaT cells at a density of 2 × 10^6^ cells/well in a six-well plate were treated with compound **1** at different concentrations or ML334 at 10 μM for 8 h. For the assessment of ROS, cells were stained with probe DCFH-DA (Beyotime, S0033s) for 0.5 h. Cells were then washed and resuspended in 1 mL phosphate buffered saline buffer and analyzed using LSRFortessa cytometry (BD Biosciences, USA). The cytometric data were analyzed with FlowJo software.

### **Luminescence intensity of compound 1**

For the assessment of luminescence intensity of compound **1**, HaCaT and A375 cells at a density of 1 × 10^6^ cells/well in a 12-well plate were treated with compound **1** at different concentrations 10 μM or 20 μM for 2 h. Cells were washed and harvested in 1 mL phosphate buffered saline (PBS) buffer and analysed using LSRFortessa cytometry (BD Biosciences, USA). The cytometric data were analysed with FlowJo software.

### **Enzyme-linked immunosorbent assay (ELISA)**

In the in vitro studies, the expressions of IL-13 in the culture supernatants obtained from the 3D RHE model were evaluated using ELISA kits (JONLNBIO, Shanghai, China) according to the manufacturer’s protocol.

### **Statistical analysis**

Statistical significance was determined using the Student's t-test for experiments comparing two groups. Comparisons among groups were analyzed using analysis of variance (ANOVA). All statistical tests were done using the GraphPad Prism version 9.0 software (GraphPad Software Inc. USA). All values were expressed as the mean ± standard deviation (SD). P < 0.05 has statistical significance.

**Table S1.** Photophysical properties of compound **1**.

| Compound | λ_em_^a^ / nm | Lifetime^a^ / ns | UV/Vis absorption^a^  λ_abs_ / nm (ɛ / dm^3^ mol^-1^ cm⁻¹) |
| --- | --- | --- | --- |
| **1** | 710 | 37.73 | 260 (0.40 × 10^5^), 328 (0.35 × 10^5^), 370 (0.26 × 10^5)^ |

Note: ^a^ means that measurements were performed in PBS buffer.

**Figure S9.** Recombinant human Keap1 protein was resolved by SDS-PAGE and visualized by Coomassie Brilliant Blue staining.

**Figure S10**. LC-MS analysis of compound **1** and Keap1. The binding between compound **1** and Keap1 protein alters the native conformation and overall hydrophobicity of the protein, resulting in a retention time (RT) shift in the chromatogram.

**Figure S11.** Luminescent response of compound **1** (10 μM) to various analytes in PBS (7.4). Compound **1** (10 μM) was incubated with 1 mM Cys and GSH, 100 μg/mL BSA, or 100 μM other interferences under ambient temperature for 0.5 h. Excitation was set at 330 nm.

**Figure S12.** (A) HaCaT cells were treated with 10 μM compound **1** for 2 h, and Cell lysates were collected and immunoblotted to detect the levels of GAPDH and Lamin B1 in the nucleus and cytoplasm. (B) The uncropped western blot images.

**Figure S13.** Effect of compound **1** or **2** in Keap1-overexpressing cells. (A) Chemical structure of compound **2**. (B) Effects of Keap1 overexpression on the imaging of compound **1** or **2** (10 μM, 2 h, **1** or **2,** λ_ex_/λ_em_ = 405/600–750 nm) in LO2 cells. Scale bar = 50 μm. (C) Western blot assay for Keap1 protein level in LO2 cell with or without Keap1 plasmid treatment. (D) Uncropped western blot images.

**Figure S14.** Confocal microscopic images of compound **1** or Lyso-tracker in HaCaT cells under continuous irradiation at 405 nm with different laser scan times (0 – 720 s). Scale bar: 25 μm.

**Figure S15**. ICP-MS quantification of 3D RHE model uptake for compound **1**. 3D RHE model were incubated with 10 or 20 µM compound **1** for 4 h. Error bars represent the standard deviations of the results from three independent experiments.

**Figure S16**. Uncropped western blot for Figure 2A (A), Figure 2D (B), Figure 3F (C), Figure 4B (D), Figure 4D (E), Figure 5B (F), Figure 6G (G).
